# Use of a capillary blood collection device to monitor exposure to per- and polyfluoroalkyl substances (PFAS) in Veterans living in proximity to potential sources of environmental contamination

**DOI:** 10.1038/s41370-026-00884-5

**Published:** 2026-04-30

**Authors:** Lauren A. Havens, Elizabeth R. Heitz, Kelsie R. Hartley, LeiAn Diaz, Jiajun Lei, Suzanne E. Fenton, Jonathan E. Katz, Andrew Patterson, Spyridoula Maraka, Terra D. Vincent-Hall, Andrew J. Morris

**Affiliations:** 1 Central Arkansas Veterans Healthcare System, Little Rock, AR USA; 2 Exposure Science Program, Health Outcomes Military Exposures (HOME), Department of Veterans Affairs, Washington, DC USA; 3 Ellison Medical Institute, Los Angeles, CA USA; 4 Center for Human Health and the Environment, Department of Biological Sciences, North Carolina State University, Raleigh, NC USA; 5 Murtha Cancer Center/Research Program, Uniformed Services University of the Health Sciences/Walter Reed National Military Medical Center, Bethesda, MD USA; 6 Department of Medicine, Keck School of Medicine, University of Southern California, Los Angeles, CA USA; 7 Eurofins Environment Testing America, West Sacramento, CA USA; 8 Division of Endocrinology and Metabolism, Department of Internal Medicine, University of Arkansas for Medical Sciences, Little Rock, AR USA; 9 Department of Pharmacology and Toxicology, University of Arkansas for Medical Sciences, Little Rock, AR USA

**Keywords:** PFAS, Environmental exposure, Remote sampling, Biomonitoring, Whole blood, Mapping.

## Abstract

**Background:**

Environmental exposure biomonitoring commonly requires a laboratory blood draw followed by processing, frozen storage, and transport of plasma or serum. Self-collection of blood onto matrices that are stable at ambient temperatures is an attractive approach for the measurement of circulating environmental chemicals.

**Objectives:**

To validate the measurement of per- and polyfluoroalkyl substances (PFAS) in capillary whole blood (WB) collected and dried onto a paper matrix using the Drawbridge Health OneDraw device. To explore sources of personal and environmental PFAS exposure in Veterans receiving care at the Central Arkansas Veterans Healthcare System (CAVHS).

**Methods:**

PFAS concentrations in dried capillary WB collected with the OneDraw device were compared to matched venous blood plasma PFAS concentrations in 133 Veterans receiving care at the CAVHS. PFAS were quantified using ultra-high-performance liquid chromatography/tandem mass spectrometry (UHPLC-MS/MS). Associations between subjects’ demographic characteristics, self-reported exposure factors, and PFAS concentrations in dried capillary WB were assessed. Geometric mean PFAS concentrations were visualized by subjects’ county of residence.

**Results:**

Measurement of 27 PFAS in dried capillary WB on OneDraw collection strips was robust with limits of quantification of 0.1 ng/mL for most analytes. In 133 Veterans, dried capillary WB levels of 10 PFAS were strongly correlated with venous blood plasma PFAS levels. WB PFAS concentrations were lower than venous blood plasma PFAS concentrations, reflecting selective partitioning of these compounds into plasma versus blood cells. WB concentrations of four PFAS (PFOA, PFOS, PFHxS, and PFNA) varied by sex and age at the time of blood draw and were associated with the primary source of drinking water.

**Significance:**

WB samples collected onto dried paper matrices, are a practical and scalable alternative to conventional venous blood draws for PFAS biomonitoring. This approach facilitates remote, repeated sampling without the need for clinical infrastructure, enabling broader participation in exposure studies.

**Impact statement:**

- Tracking environmental exposures in military, occupational, and community contexts has been challenging, particularly due to the lack of a practical workflow for capturing biological samples without a clinical infrastructure. This has led to a reduced ability to capture personal exposure data, in turn, limiting the analyses of the impact of these exposures on long-term health. We developed and validated a robust method using a self-contained, blood collection device for PFAS exposure biomonitoring as a proof-of-concept for broader adoption of this approach to enable larger-scale, longitudinal studies of exposure to PFAS and other classes of environmental chemicals. This method can be used to improve the evaluation of exposure-outcome relationships and provides a foundation for evidence-based policy decisions for health care and benefits.

## Introduction

Per- and polyfluoroalkyl substances (PFAS) are synthetic chemicals with widespread applications in consumer products and industrial processes. Among its other uses, PFAS are components of aqueous film-forming foams (AFFF) used for fighting hydrocarbon fuel fires in military and occupational settings [1]. In addition to potential occupational exposure, the storage and use of AFFF on military bases and fire-training centers have contributed to widespread groundwater and drinking water contamination, potentially impacting both military personnel and civilians living in nearby communities [2]. Some PFAS are highly persistent in the body [3], and exposure has been associated with a range of deleterious health outcomes, including endocrine and immune system dysfunction, hyperlipidemia and cardiometabolic diseases, and certain types of cancer [4, 5].

Environmental exposures, including to persistent chemicals like PFAS, are important contributors to human disease risk [6, 7]. While environmental exposures can have long-term impacts on health, windows of opportunity for exposure biomonitoring are often relatively short. Measurements of circulating levels of environmental chemicals generally involve a venous blood draw in a clinic with subsequent sample processing, cryostorage, and local or remote analytical measurements [8]. These logistical demands often limit the feasibility of collecting individual-level exposure data in the timeframe in which some compounds would be observable [9]. As a result, many environmental exposure assessments rely on indirect estimates, which reduce their precision and limit their usefulness for evaluating health risks. Due to their long biological persistence and relevance to health, PFAS provide an opportunity to develop and validate a workflow for exposure analysis that allows for rapid deployment, supports biobanking, and does not depend on a clinical infrastructure.

Accurate, reproducible methods for quantification of panels of PFAS, including common constituents of AFFF, have been widely reported and validated in the literature. These methods generally employ UHPLC-coupled electrospray ionization tandem mass spectrometry (MS) with triple quadrupole instruments [8]. Using these methods, PFAS can be detected at blood concentrations in the range of 0.1-10 ng/mL, commonly observed in plasma or serum of essentially all adult Americans [10]. Although circulating PFAS levels in individuals with higher exposure often exceed 100 ng/mL, these assays are well-suited to detect individuals at the 20 ng/mL threshold, which is suggested to warrant enhanced clinical care and follow-up [4].

The aim of this study was to develop methods for PFAS measurement in dried capillary whole blood (WB) and to compare those values to venous blood from the same individuals. Validation of this methodology will enable a practical workflow for capturing and measuring environmental exposures in biological samples without a clinical infrastructure. This will allow for capturing personal exposure data and assessing the impact of those exposures on long-term health [11]. This is especially important for individuals working in certain occupational settings, such as firefighting, emergency response, and military deployments.

## Materials and methods

### Study participants

**Fig. 1:**
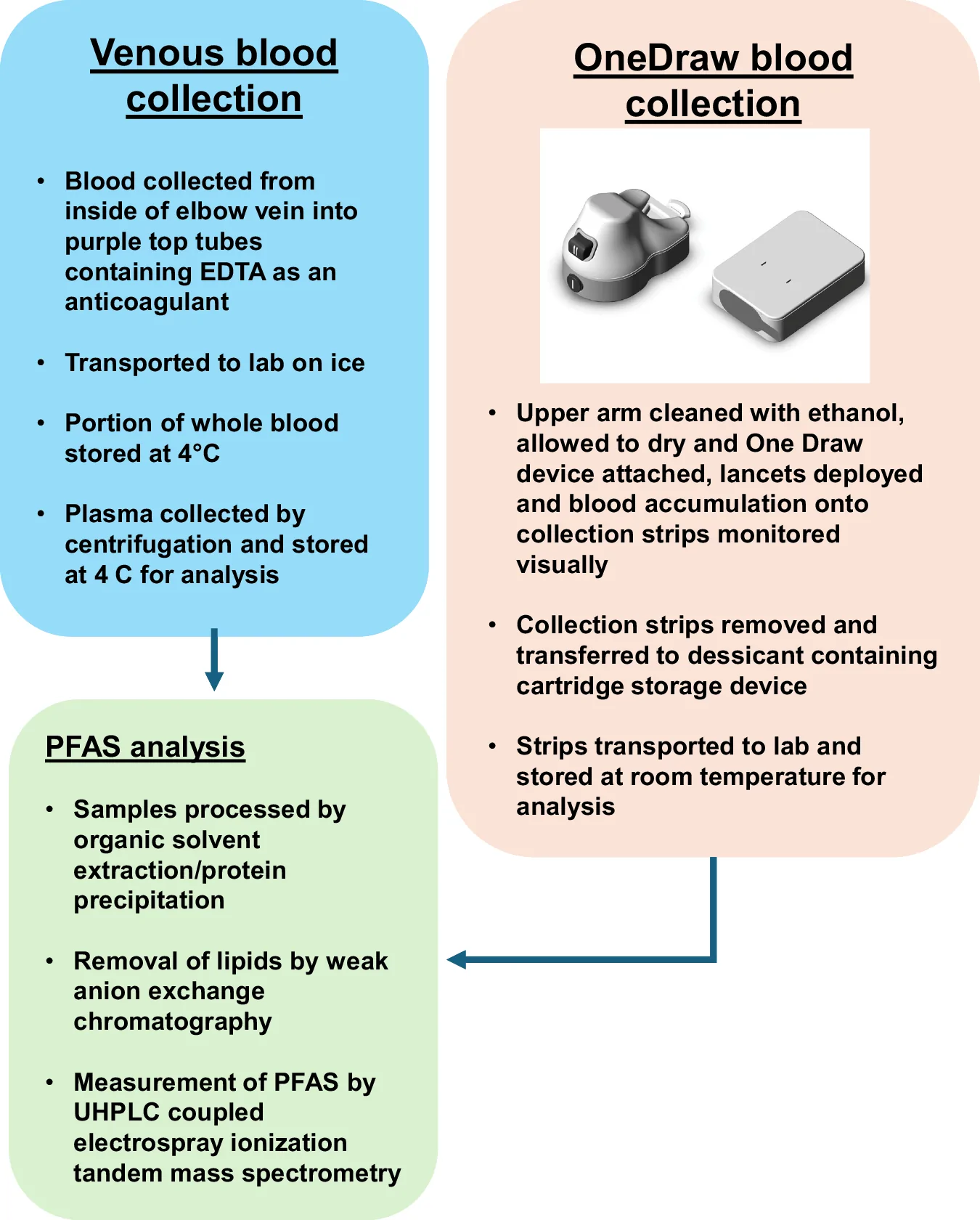
Study overview, sample collection and analysis workflow.

Veterans receiving outpatient care at the John L. McClellan Memorial Veterans Hospital in Little Rock, AR, provided written informed consent and human blood samples were collected between December 2023 and October 2024. Veterans with diagnosed cancer, kidney, liver, cardiovascular, and thyroid diseases, and dementia were excluded from the study. Figure 1 shows an overview of the study design. The protocol was approved by the Central Arkansas Veterans Healthcare System Institutional Review Board. All subjects provided written informed consent.

### Sample collection

After wiping the skin with an alcohol swab, capillary blood from the upper arm was collected using the OneDraw device with assistance from a clinical study coordinator and stored at room temperature inside the provided desiccant-containing transport cassette until the time of testing per vendor instruction, resulting in two dried blood strips (DBS) for each subject. Time-matched venous blood draws from the veins in the antecubital fossa (anterior side of the elbow) were also collected by a phlebotomist in 4-mL purple top collection tubes before sub-aliquoting into polypropylene centrifuge tubes (0.5 mL each) and centrifuging at 2000 × *g* for 10 min. The supernatant plasma fractions were transferred into secondary tubes and frozen at –18 °C in 0.25 mL fractions, while the remaining whole blood fractions (approximately 3 mL) were refrigerated at 4 °C.

In total, we enrolled 200 Veterans into the study. In 67 cases, the OneDraw device strips were underfilled. The study cohort, therefore, generated matched venous plasma and completely filled OneDraw samples from 133 Veterans. Each participant also completed a brief questionnaire at the time of sample collection that included questions about possible sources of PFAS exposure, such as occupational history, primary source of drinking water, and all cities and states of residence in the past five years. The full questionnaire is included as Supplemental Document 1.

Complete details of the materials used in this study, sample collection, quality control assessment, sample preparation, solid phase extraction, calibration curves, LC-MS data acquisition, processing and reporting, and statistical and geospatial analysis are provided as Supplemental Document 2.

## Results

### Participant characteristics

One hundred and thirty-three Veterans concurrently provided both capillary DBS and venous blood samples (Table 1). These participants were predominantly white (63.2%) and male (71.4%). They ranged in age from 34 to 80, with 74.5% over the age of 54. Over 50% of participants lived in Pulaski County, where the CAVHS is located; however, many reported having lived outside the county within the past 5 years (data not shown). Most participants reported that municipal systems were their primary source of drinking water (54.1%), never having performed firefighting duties (75.2%), and not having donated blood in the past twelve months (94%). Finally, self-report of exposure to stain/water repellent or not was similar (46.6% vs. 44.4%, respectively).

**Table 1:** Study Population Characteristics.

Characteristic	Participants (*N* = 133)
*Demographics*	
**Age**	
Under 40 years	6.0% (8)
40 to 54 years	19.6% (26)
55 to 69 years	42.9% (57)
70 years and over	31.6% (42)
**Sex**	
Male	71.4% (95)
Female	28.6% (38)
**Race and ethnicity**	
White, non-Hispanic	63.2% (84)
Black or African American, non-Hispanic	36.1% (48)
White, Hispanic	0.8% (1)
**County of residence**	
Faulkner	9.8% (13)
Lonoke	12.0% (16)
Pulaski	54.9% (73)
Saline	15.0% (20)
Other, including out of state	8.3% (11)
*Self-reported exposure factors*	
**Ever used AFFF for firefighting while in the military**	
Yes	17.3% (23)
No, but involved in firefighting	7.5% (10)
No, never involved in firefighting	75.2% (100)
**Primary source of drinking water**	
Municipal	54.1% (72)
Bottled	39.9% (53)
Private well	3.0% (4)
Do not know or not reported	3.0% (4)
**Exposure to stain repellent or water protective coatings**	
Yes	46.6% (62)
No	44.4% (59)
Do not know	9.0% (12)
**Donated blood in the past 12 months**	
Yes	6.0% (8)
No	94.0% (125)

### Analytical method validation and data reporting

Data from plasma and DBS method validation are summarized in Table 2 and Supplemental Spreadsheet 1. Table 2 shows the limits of quantification (LOQs) determined in the validation studies for both plasma and DBS matrices. If an analyte was unable to be validated in a particular matrix, it is marked not validated (NV). Additionally, analytes that did not meet reporting requirements as previously described were not reported (NR). For example, PFBA and PFPeA were frequently detected at concentrations outside the acceptance criteria in the matrix blank and double blank; therefore, levels of these analytes in participant samples were not reported in this study.

**Table 2:** Frequency of detection and concentration range of all measured PFAS in plasma and OneDraw whole dried blood.

Analyte Abbreviation	Limit of Quantification (ng/mL)		Frequency (%)		Range (ng/mL)	
	Plasma	DBS	Plasma	DBS	Plasma	DBS
PFBS	0.1	0.1	0	0	–	–
PFPES	NV	0.1	–	0	–	–
PFHxS L	0.08	0.08	97.74	89.47	0.08-3.40	0.08-1.86
PFHxS Br	0.1	0.1	58.65	24.06	0.1-0.91	0.1-0.43
**PFHxS Total**	**0.1**	**0.1**	**99.25**	**91.73**	**0.1-3.96**	**0.1-2.24**
PFHpS	0.1	0.1	54.89	15.04	0.1-0.56	0.1-0.32
PFOS L	0.08	0.08	100	98.50	0.08-16.83	0.1-5.91
PFOS Br	0.1	0.1	98.50	93.23	0.1-11.78	0.1-5.26
**PFOS Total**	**0.1**	**0.1**	**100**	**98.50**	**0.16-22.76**	**0.1-8.81**
PFNS	0.1	0.1	0.75	0	0.1-0.59	–
PFDS	0.1	NV	0	0	–	–
PFBA	0.3	0.3	NR	NR	–	–
PFPeA	0.1	0.1	NR	NR	–	–
PFHxA	0.1	0.3	0	0	–	–
PFHpA	0.1	0.1	0.75	0	0.1-0.13	–
**PFOA**	**0.1**	**0.1**	**98.50**	**92.48**	**0.1-2.64**	**0.1-1.03**
**PFNA**	**0.1**	**0.1**	**90.98**	**42.86**	**0.1-0.39**	**0.1-0.36**
PFDA	0.1	0.1	43.61	15.79	0.1-1.47	0.1-0.71
PFUdA	0.1	0.1	10.53	7.52	0.1-0.45	0.1-0.19
PFDoA	0.1	0.1	1.50	0	0.1-0.19	–
PFTrDA	0.1	NV	0	NR	–	–
PFTeDA	0.1	0.3	0	0	–	–
**HFPO-DA**	**0.1**	**0.1**	**0**	**2.26**	–	**0.1-0.12**
NaDONA	0.3	0.3	0	0	–	–
FOSA	0.3	0.3	0	14.29	–	0.1-3.67
N-MeFOSAA L	0.08	0.08	15.79	6.02	0.1-1.86	0.1-0.91
N-MeFOSAA BR	0.1	0.1	3.76	0.75	0.1-0.58	0.1-0.42
N-MeFOSAA Total	0.1	0.1	21.05	6.02	0.1-2.44	0.1-1.33
N-EtFOSAA L	0.08	0.08	0	0	–	–
N-EtFOSAA Br	0.1	0.1	0	0	–	–
N-EtFOSAA Total	0.1	0.1	0	0	–	–
4:2 FTS	0.1	0.3	0	0	–	–
6:2 FTS	10	10	0	0	–	–
8:2 FTS	0.1	0.1	0	0	–	–
9CI-PF3ONS	25	0.1	0	0	–	–
FBSA	10	0.3	0	0	–	–
FHxSA	NV	20	-	0	–	–

NV-assay not validated, NR-not reported (see methods for details). PFAS with the U.S. Environmental Protection Agency (EPA) drinking water maximum contamination levels are in bold.

### Comparison of plasma and DBS PFAS levels

The range of concentrations for all measured PFAS in matched plasma and DBS is shown in Table 2. For the current study, certain PFAS were targeted in subsequent analyses. These analytes were detected at the following ranges: PFHxS: 0.1–3.96 (plasma), 0.1–2.24 (DBS); PFOS: 0.16–22.76 (plasma), 0.1–8.81 (DBS); PFOA: 0.1–2.64 (plasma), 0.1–1.03 (DBS); and PFNA: 0.1–0.94 (plasma), 0.1–0.36 (DBS).

Frequencies of detection for each analyte are also reported in Table 2. In general, concentrations of PFAS in DBS are approximately 48% of those in plasma. Therefore, subjects with PFAS concentrations close to the LOQ in plasma had corresponding measurements in DBS that were below the LOQ. Detection frequencies for PFOA, PFOS and PFHxS were >90% in both plasma and DBS, as detected levels were well above the LOQ. Conversely, the detection frequency for PFNA was 91% in plasma but only 43% in DBS, as detected levels in plasma were often close to the LOQ.

Some PFAS analytes, such as HFPO-DA, were rarely detected in participants. PFBS was not detected in any of the plasma or DBS samples. PFAS analytes that were not consistently detected in the study participants were not included in further analyses.

**Fig. 2:**
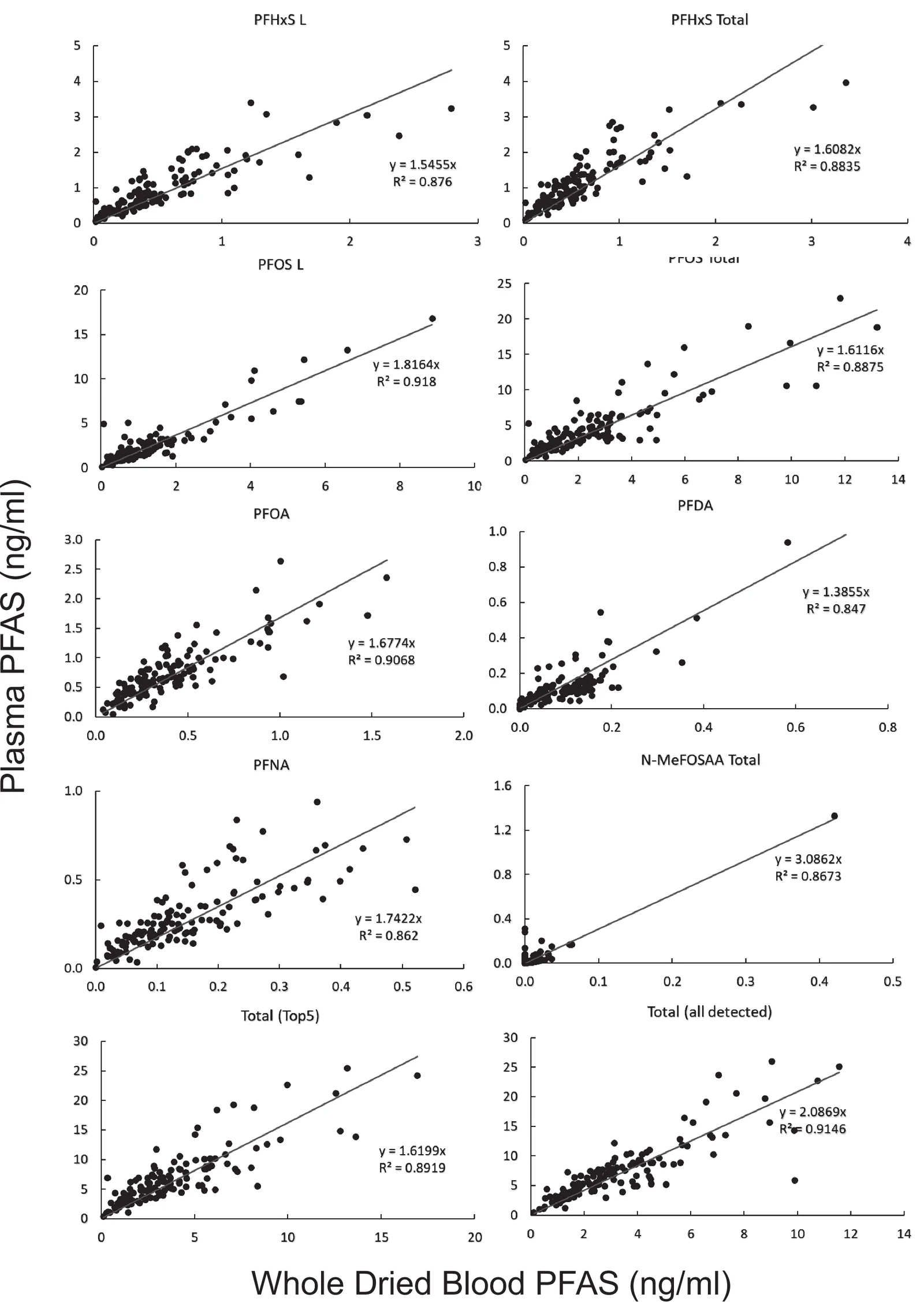
Relationships between venous blood plasma and OneDraw collected whole dried capillary blood PFAS concentrations. Scatter plots are presented for the indicated PFAS or mixtures of PFAS. The data were analyzed by linear regression, and the regression constants (R2) and coefficients are shown.

Correlations between plasma and DBS PFAS concentrations are shown in Fig. 2. Pearson’s and Spearman’s rank correlation coefficients revealed very strong (*p* < 0.001), positive correlations between plasma and DBS levels of several PFAS analytes, including PFHxS (0.92 and 0.93, respectively), PFHpS (0.81 and 0.86), PFOS (0.92 and 0.88), PFOA (0.93 and 0.92), PFNA (0.85 and 0.87), PFDA (0.94 and 0.83), PFUdA (0.86 and 0.77), and N-MeFOSAA (0.88 and 0.62). Regression constants were >0.90 for PFOS, PFHxS, PFOA and N-MeFOSAA and >0.85 for PFDA and PFNA. The regression constant for the sum of the four PFAS used for subsequent analyses (PFOA, PFOS, PFHxS and PFNA) was 0.89. For all detected PFAS, it was 0.90. In all cases, *p*-values were <0.001, indicating a statistically significant relationship between the predictor (DBS) and response (plasma) variables.

### Demographic characteristics associated with PFAS body burden

Table 3 shows geometric mean (GM) levels of the four target PFAS (PFOS, PFOA, PFHxS, and PFNA) in DBS by selected characteristics of the study population. Our DBS data revealed that one hundred and thirty-two Veterans (99.2%) had measurable circulating PFAS levels above the reporting level for at least one of the target PFAS analytes, which accounted for the majority of circulating PFAS in all subjects. PFOA was detected in 92.5% of Veterans, PFOS in 98.4%, PFHxS in 91.7%, and PFNA in 42.9%. An estimated concentration of 0.1/sqrt(2) (approximately 0.07) was imputed for Veterans with levels below the limit of quantification for each analyte (e.g., 7.5% of PFOA values and 57.5% of PFNA values) prior to calculating GMs. GM concentration of total target PFAS in DBS was 2.20 (95% confidence interval (CI) = 1.95–2.48), largely driven by PFOS levels (N_detect_=131, GM = 1.36, 95% CI = 1.18–1.57). Circulating PFAS concentrations varied significantly by participant age. Detected levels of total target PFAS increased with age (Over 70 years: *N* = 42, GM = 2.85, 95% CI = 2.32–3.49; Under 40 years: *N* = 8, GM = 1.36, 95% CI = 0.85–2.17; *p* = 0.0036). Male sex was also associated with higher circulating total target PFAS compared to female (Males: *N* = 95, GM = 2.50, 95% CI = 2.19–2.87; Females: *N* = 38, GM = 1.59, 95% CI = 1.28–1.97; *p* = 0.0006). Table 4 shows the same information for the target PFAS in matched plasma samples from the same subjects. Detection frequencies for four of the five PFAS are marginally higher in plasma because the effective concentration of PFAS is higher in plasma than in whole blood. Specifically, total PFAS was detected in DBS from 132 subjects, but this increased to 133 subjects when plasma was analyzed.

**Table 3:** Associations between DBS PFAS concentrations and selected study population characteristics.

	EPA Regulated PFAS^1^ (N_detect_=132)		PFOA (N_detect_=123)		PFOS^2^ (N_detect_=131)		PFHxS^3^ (N_detect_=122)		PFNA (N_detect_=57)	
	GM (95% CI)^4^	*P*^*5*^	GM (95% CI)	*P*	GM (95% CI)	*P*	GM (95% CI)	*P*	GM (95% CI)	*P*
**Total**	2.38 (2.13–2.66)		0.25 (0.23–0.28)		1.36 (1.18–1.57)		0.38 (0.34–0.43)		0.10 (0.09–0.11)	
**Age**										
Under 40 years	1.52 (0.99–2.35)	0.0034	0.22 (0.14–0.34)	0.2336	0.73 (0.42–1.28)	0.0014	0.30 (0.19–0.48)	0.3250	0.08 (0.05–0.11)	0.0217
40 to 54 years	1.86 (1.46–2.37)		0.25 (0.20–0.33)		0.96 (0.70–1.30)		0.34 (0.27–0.45)		0.08 (0.07–0.10)	
55 to 69 years	2.37 (2.02–2.79)		0.23 (0.19–0.27)		1.38 (1.12–1.70)		0.37 (0.31–0.44)		0.11 (0.10–0.12)	
70 years and over	3.02 (2.50–3.65)		0.29 (0.24–0.35)		1.86 (1.46–2.37)		0.44 (0.36–0.54)		0.11 (0.10–0.13)	
**Sex**										
Male	2.69 (2.37–3.05)	0.0004	0.26 (0.23–0.29)	0.4627	1.56 (1.32–1.84)	0.0023	0.45 (0.39–0.51)	<.0001	0.11 (0.10–0.12)	0.0608
Female	1.75 (1.44–2.14)		0.24 (0.19–0.29)		0.96 (0.74–1.25)		0.25 (0.21–0.31)		0.09 (0.08–0.11)	
**Race**										
White^6^	2.35 (2.05–2.70)	0.8026	0.28 (0.25–0.32)	0.0046	1.30 (1.09–1.55)	0.4000	0.41 (0.35–0.47)	0.0830	0.10 (0.09–0.11)	0.3358
Black or African American	2.42 (2.01–2.92)		0.20 (0.17–0.24)		1.47 (1.16–1.87)		0.33 (0.27–0.40)		0.11 (0.09–0.12)	
**Used AFFF for firefighting while in the military**										
Yes	2.60 (1.99–3.40)	0.5208	0.29 (0.22–0.37)	0.5001	1.53 (1.08–2.16)	0.4740	0.42 (0.32–0.55)	0.7346	0.11 (0.09–0.14)	0.4708
No, but involved in firefighting	2.77 (1.85–4.16)		0.25 (0.17–0.38)		1.69 (1.01–2.85)		0.39 (0.26–0.60)		0.11 (0.08–0.15)	
No, never involved in firefighting	2.29 (2.02–2.61)		0.24 (0.21–0.27)		1.29 (1.10–1.53)		0.37 (0.32–0.42)		0.10 (0.09–0.11)	
**Primary source of drinking water**										
Municipal	2.62 (2.26–3.03)	0.0278	0.27 (0.24–0.31)	0.0323	1.54 (1.27–1.86)	0.0226	0.41 (0.35–0.48)	0.1055	0.10 (0.09–0.12)	0.0551
Bottled	2.05 (1.73–2.44)		0.22 (0.19–0.26)		1.13 (0.91–1.42)		0.33 (0.28–0.40)		0.10 (0.08–0.11)	
Private well	4.15 (2.22–7.76)		0.40 (0.22–0.74)		2.66 (1.19–5.95)		0.66 (0.34–1.26)		0.17 (0.11–0.28)	
Do not know	1.46 (0.71–3.02)		0.13 (0.06–0.26)		0.61 (0.24–1.55)		0.33 (0.15–0.69)		0.07 (0.04–0.12)	
**Exposure to stain repellent or water protective coatings**										
Yes	2.45 (2.07–2.89)	0.6533	0.30 (0.25–0.35)	0.0113	1.36 (1.09–1.68)	0.7222	0.42 (0.35–0.49)	0.0962	0.10 (0.09–0.12)	0.8497
No	2.39 (2.03–2.81)		0.23 (0.20–0.27)		1.41 (1.14–1.74)		0.37 (0.32–0.44)		0.10 (0.09–0.11)	
Do not know	2.03 (1.40–2.93)		0.18 (0.13–0.26)		1.14 (0.71–1.84)		0.26 (0.18–0.39)		0.10 (0.08–0.14)	
**Donated blood in the past 12 months**										
Yes	1.84 (1.17–2.88)	0.2436	0.17 (0.11–0.27)	0.0881	0.98 (0.55–1.76)	0.2556	0.38 (0.23–0.60)	0.9637	0.09 (0.06–0.12)	0.3733
No	2.42 (2.16–2.71)		0.26 (0.23–0.29)		1.39 (1.20–1.61)		0.38 (0.34–0.43)		0.10 (0.09–0.11)	

^1^Total blood concentration including 6 PFAS currently regulated by the U.S Environmental Protection Agency (EPA): PFOA, PFOS, PFHxS, PFNA, HFPO-DA, and PFBS.

^2^Includes both linear (n-PFOS) and branched (br-PFOS) isomers measured separately.

^3^Includes both linear (n-PFHxS) and branched (br-PFHxS) isomers measured separately.

^4^Geometric mean (GM) and 95% confidence interval (CI).

^5^*p-value* for t-test or ANOVA, difference between groups considered significant at α = 0.05.

^6^Includes Hispanic ethnicity.

**Table 4:** Associations between plasma PFAS concentrations and selected study population characteristics.

	Total Target PFAS^1^ (N_detect_=133)^2^		PFOA (N_detect_=131)^2^		PFOS (N_detect_=133)^2^		PFHxS (N_detect_=132)^2^		PFNA (N_detect_=57)^2^	
	GM (95% CI)^3^	*P*^*4*^	GM (95% CI)^3^	*P*^*4*^	GM (95% CI)^3^	*P*^*4*^	GM (95% CI)^3^	*P*^*4*^	GM (95% CI)^3^	*P*^*4*^
**Total**	5.34 (4.76–5.99)		0.61 (0.55–0.68)		3.33 (2.91–3.81)		0.90 (0.80–1.02)		0.23 (0.20–0.26)	
**Age**										
Under 40 years	3.69 (2.37–5.76)	0.0009	0.46 (0.29–0.72)	0.1298	2.15 (1.29–3.60)	0.0005	0.77 (0.47–1.24)	0.1980	0.13 (0.08–0.22)	0.0028
40 to 54 years	3.78 (2.95–4.84)		0.57 (0.44–0.73)		2.15 (1.61–2.86)		0.76 (0.58–1.00)		0.16 (0.12–0.22)	
55 to 69 years	5.42 (4.59–6.41)		0.57 (0.48–0.68)		3.49 (2.87–4.23)		0.88 (0.73–1.05)		0.24 (0.20–0.29)	
70 years and over	6.96 (5.73–8.45)		0.73 (0.60–0.89)		4.47 (3.57–5.59)		1.07 (0.87–1.32)		0.29 (0.23–0.36)	
**Sex**										
Male	6.11 (5.37–6.96)	0.0002	0.62 (0.54–0.70)	0.8060	3.88 (3.34–4.51)	0.0003	1.06 (0.92–1.20)	<.0001	0.24 (0.20–0.27)	0.3734
Female	3.82 (3.11–4.69)		0.60 (0.48–0.74)		2.28 (1.79–2.89)		0.61 (0.50–0.75)		0.21 (0.16–0.26)	
**Race**										
White^5^	5.17 (4.47–5.97)	0.4456	0.66 (0.58–0.76)	0.0622	3.07 (2.60–3.62)	0.1065	0.97 (0.84–1.13)	0.0991	0.22 (0.19–0.25)	0.3578
Black or African American	5.67 (4.68–6.87)		0.53 (0.44–0.64)		3.85 (3.09–4.81)		0.79 (0.65–0.96)		0.25 (0.20–0.30)	
**Used AFFF for firefighting while in the military**										
Yes	5.99 (4.55–7.90)	0.3484	0.71 (0.54–0.92)	0.4955	3.75 (2.72–5.17)	0.2908	1.01 (0.76–1.35)	0.5961	0.26 (0.19–0.36)	0.5861
No, but involved in firefighting	6.57 (4.32–10.00)		0.62 (0.41–0.94)		4.47 (2.75–7.28)		0.99 (0.64–1.53)		0.24 (0.15–0.38)	
No, never involved in firefighting	5.10 (4.46–5.82)		0.59 (0.52–0.67)		3.15 (2.70–3.67)		0.87 (0.76–1.00)		0.22 (0.19–0.25)	
**Primary source of drinking water**										
Municipal	5.84 (4.99–6.82)	0.2404	0.66 (0.57–0.77)	0.3297	3.68 (3.07–4.41)	0.2538	0.98 (0.83–1.15)	0.3482	0.25 (0.21–0.30)	0.2632
Bottled	4.63 (3.86–5.56)		0.55 (0.46–0.66)		2.83 (2.29–3.49)		0.80 (0.66–0.96)		0.19 (0.16–0.24)	
Private well	6.85 (3.53–13.29)		0.67 (0.35–1.28)		4.50 (2.09–9.72)		1.22 (0.61–2.42)		0.28 (0.13–0.58)	
Do not know	4.93 (2.30–10.59)		0.42 (0.20–0.89)		3.23 (1.33–7.86)		0.86 (0.39–1.91)		0.19 (0.08–0.45)	
**Exposure to stain repellent or water protective coatings**										
Yes	5.38 (4.52–6.40)	0.9712	0.71 (0.60–0.84)	0.0557	3.19 (2.61–3.91)	0.7704	0.97 (0.81–1.16)	0.2448	0.24 (0.20–0.29)	0.3323
No	5.27 (4.45–6.25)		0.54 (0.46–0.63)		3.38 (2.78–4.12)		0.90 (0.75–1.06)		0.21 (0.17–0.25)	
Do not know	5.53 (3.76–8.13)		0.56 (0.39–0.82)		3.79 (2.42–5.93)		0.67 (0.45–0.99)		0.28 (0.19–0.43)	
**Donated blood in the past 12 months**										
Yes	4.22 (2.64–6.75)	0.3081	0.38 (0.24–0.60)	0.0372	2.56 (1.49–4.42)	0.3290	0.87 (0.53–1.41)	0.8719	0.15 (0.09–0.25)	0.0970
No	5.42 (4.82–6.11)		0.63 (0.56–0.71)		3.39 (2.95–3.89)		0.91 (0.80–1.02)		0.23 (0.20–0.27)	

^1^Total blood concentration including 4 PFAS of interest: PFOA, PFOS, PFHxS, and PFNA. HFPD-OA and PFBS are not included in this total due to the low numbers of participants with

plasma concentrations above the limit of detection (LOD) (N_detect_ HFPO-DA = 0, N_detect_ PFBS = 0).

^2^All participants (*N* = 133) were included in all models. For each analyte, DBS concentrations below the LOD were imputed as a value equal to the LOD divided by the square root of 2.

^3^Geometric mean (GM) and 95% confidence interval (CI).

^4^*p-value* for t-test or ANOVA, difference between groups considered significant at α = .05.

^5^Includes Hispanic ethnicity (*N* = 1).

### Exposure factors associated with PFAS body burden

As shown in Table 3, the primary source of drinking water was significantly associated with total target PFAS (*p* = 0.0249). Levels were highest in Veterans who reported primarily drinking private well water (*N* = 4, GM = 3.97 ng/mL, 95% CI = 2.02–7.81), followed by municipal water (*N* = 72, GM = 2.44 ng/mL, 95% CI = 2.08–2.87), bottled water (*N* = 53, GM = 1.88 ng/mL, 95% CI = 1.56–2.26), and “do not know” (*N* = 4, GM = 1.24 ng/mL, 95% CI = 0.57–2.72). A similar pattern was observed in the individual target PFAS, though only variations in PFOA and PFOS by drinking water source reached significance (*p* = 0.0323 and *p* = 0.0226, respectively).

Total target PFAS levels were similar for those who self-reported exposure to stain repellent or water protective coatings compared to those who did not (*p* = 0.5792). However, self-reporting these exposures was associated with circulating PFOA levels (*N* = 62, GM = 0.30 ng/mL, 95% CI = 0.25–0.35). Exposure to stain repellent or water protective coatings was not associated with differences in any other individual target PFAS.

Blood donation in the past 12 months and involvement in firefighting while in the military were also evaluated as exposure factors. The geometric mean for total target PFAS appeared somewhat lower among Veterans who reported donating blood in the past 12 months (*N* = 8, GM = 1.67 ng/mL, 95% CI = 1.03–2.72) compared to Veterans who did not (*N* = 125, GM = 2.24 ng/mL, 95% CI = 1.98–2.53), although the difference was not statistically significant (*p* = 0.2542). PFAS body burden was similar in Veterans who participated in military firefighting, with or without exposure to AFFF, and those with no military firefighting involvement (*p* = 0.5095 for total target PFAS).

## Discussion

This study demonstrated the feasibility of measuring multiple PFAS in whole blood on DBS collected via the OneDraw device. In most cases, assay performance was comparable to that observed when analyzing blood plasma. Further, DBS PFAS levels correlated strongly with and were predictive of plasma PFAS levels. Our findings suggest that the complement of PFAS in venous blood plasma is comparable to that of extracts from capillary DBS.

Plasma PFAS levels were consistently higher than in whole blood extracted from matched DBS. This likely reflects the distribution of these compounds exclusively in the plasma component of blood, as has been reported by others [12], with the exception of FOSA, which has been reported to have higher detected concentrations in whole blood compared to plasma [13]. Adjustments for interindividual differences in hematocrit may make whole blood PFAS measurements more comparable to plasma or serum measurements. However, since the biological range of hematocrit in otherwise healthy individuals is relatively narrow, for screening purposes, adjusting by the gender-average hematocrit value may be sufficient. The higher effective concentration of PFAS in plasma resulted in marginally higher detection frequencies for some PFAS in plasma vs DBS at lower exposure levels. This small increase in detection frequency did not impact our analysis of associations between PFAS levels and selected participant characteristics, further supporting the value of DBS for PFAS exposure assessment.

PFAS levels in our study cohort are broadly comparable to the general US adult population [10, 14]. Consistent with many other studies, circulating PFAS levels were significantly higher in older versus younger individuals, potentially reflecting the persistent nature of these chemicals and their slow elimination from the body through mechanisms that may be less efficient or nonexistent in older individuals [15–17]. As widely observed in other cohorts, male sex was strongly associated with higher circulating PFAS levels compared to females [14, 18]. This sex-related difference in PFAS body burden is likely related to females of child-bearing age being able to eliminate PFAS through mechanisms that are not feasible for males (i.e., menstruation, breastfeeding) [19].

In the present study, PFAS body burden varied by some self-reported exposure factors. A small number of Veterans who primarily drink private well water had high circulating levels of target PFAS. Although several national studies have detected PFAS less frequently in private wells compared to public water systems [20, 21], regulation of private wells is limited [22], and their owners may not routinely test for PFAS [23, 24]. We did not assess the specific locations of these private wells; therefore, it is possible that their siting places them at high risk for contamination (e.g., close to industrial or agricultural exposure sources). Alternatively, the increased burden of PFAS in private well water users may be related to uncontrolled confounding factors, such as residence in rural, agricultural areas with widespread spraying of crops with pesticides containing PFAS, which are added to improve stability and dispersion[25]. Personal exposure to stain-repellent or water protective coatings was also associated with variation in circulating PFOA, with the highest levels observed in Veterans who reported having been exposed. In both analyses, Veterans who reported that they “do not know” what their primary source of drinking water is or whether they were exposed to repellent or protective coatings had the lowest DBS PFAS levels. This study did not directly assess PFAS-risk perception; however, it is possible that these participants’ lack of awareness about PFAS exposure factors may be related to the fact that they have never knowingly been exposed [26].

Other exposure factors were not associated with variation in DBS PFAS levels. Though AFFF is a well-known source of occupational and environmental PFAS exposure [1, 2], involvement in firefighting while in the military, including the use of AFFF, was not associated with any differences in PFAS body burden in this study. This finding may be related to the fact that all participants were Veterans, and any prior military exposures were less relevant than recent environmental exposures. Alternatively, this finding could be related to how participants interpreted and responded to the question (Supplemental Document 1). Veterans who reported a history of being involved in military firefighting included those with less than one year of experience up to ten years of experience (data not shown). Though a shorter duration of firefighting duties would likely result in less cumulative exposure to AFFF, we did not attempt to parse these data due to already small sample sizes. In this study, circulating total target PFAS appeared to be slightly lower in Veterans who reported donating blood in the past 12 months, but the difference did not reach statistical significance. This finding was not consistent with previously published observations that blood and plasma donation reduced circulating PFAS burden [27]. The finding in the current study may have been due to the small number of people who reported donating blood or to some unmeasured confounder.

Findings from the current study were consistent with several published studies using fingerstick whole blood collected onto filter paper or using gel-based microsamplers for PFAS exposure biomonitoring [13, 28–31]. In general, PFAS were able to be extracted from the DBS at levels that were usually above the LOQ. The high correspondence of venous plasma and capillary whole blood measurements shown in this study establishes that DBS collection can be an effective, integrated system for whole blood collection, storage, and subsequent analysis, which may be supportive of a broad range of environmental chemical exposure biomonitoring.

Removal of lipids during sample preparation is essential for sensitive, robust PFAS measurements. This is most often accomplished using anion-exchange chromatography to separate bound PFAS from the bulk lipids in biological matrices, which are generally cationic or neutral [8]. Interestingly, using MS-based lipidomics, lipids in all major classes were significantly retained on the OneDraw strips under the conditions used for efficient extraction of PFAS (data not shown). This binding of lipids to filter paper has been reported previously and likely involves hydrophobic binding of the lipid hydrocarbon chains to cellulose [32]. This phenomenon has been exploited for the removal of lipids from biological matrices for metabolite measurements [33]. Retention of lipids on OneDraw strips might offer benefits for measurements of other classes of environmental chemicals in OneDraw-collected DBS while still providing an opportunity for lipid analysis using more aggressive extraction protocols. Indeed, it is likely that this collection method could be effective for exposure biomonitoring of other classes of polar environmental chemicals. Whole blood measurements might offer superior sensitivity for analysis of less polar, lipophilic compounds that preferentially associate with the cellular components of whole blood and include many classes of environmental chemicals of concern.

In summary, our results show that the capillary blood collection is sufficient for PFAS exposure biomonitoring. Although collection occurred under the supervision of a study coordinator in this study, similar devices can be provided to individuals who can collect their own blood. Filled strips can then be mailed or transported to a laboratory for subsequent analysis. This could obviate the need for a clinical visit for blood drawing, immediate processing and storage. Self-collection of whole dried blood could enable real-time exposure biomonitoring in at-risk individuals (for instance, following military deployment or a fire event). This approach could also facilitate repeated sampling for longitudinal exposure studies.

## Supplementary information

## Data Availability

Primary data are available on request.
