# Supplementary material for: Use of a capillary blood collection device to monitor exposure to per- and polyfluoroalkyl substances (PFAS) in Veterans living in proximity to potential sources of environmental contamination
Source: J Expo Sci Environ Epidemiol. 2026 Apr 30;36(5):878–87. doi: 10.1038/s41370-026-00884-5 (PMC13538070; doi:10.1038/s41370-026-00884-5)
Supplement: Supplementary file 1 [file 41370_2026_884_MOESM1_ESM.docx]

**Supplemental Materials**

**Supplemental Table 1**. PFAS names, CASRNs and abbreviations.

**Supplemental Table 2**. Mass spectrometer operating parameters and settings.

**Supplemental Table 3.** Data sources for geospatial analysis.

**Supplemental Spreadsheet 1.** Complete assay validation dataset.

**Supplemental Document 1.** Supplemental methods

**Supplemental Document 2.** Study subject questionnaire

**Supplemental Document 3** Geospatial analysis results and discussion.
