# Supplementary material for: Use of a capillary blood collection device to monitor exposure to per- and polyfluoroalkyl substances (PFAS) in Veterans living in proximity to potential sources of environmental contamination
Source: J Expo Sci Environ Epidemiol. 2026 Apr 30;36(5):878–87. doi: 10.1038/s41370-026-00884-5 (PMC13538070; doi:10.1038/s41370-026-00884-5)
Supplement: Supplementary file 2 [file 41370_2026_884_MOESM2_ESM.docx]

**Purpose:** This information will be used to identify possible sources of PFAS exposure over the last 12 months and activities that may have had an impact on the PFAS levels in your blood.

**Instructions**: You may choose to skip any question. Please answer the questions to the best of your ability. Thank you for taking the time to complete this questionnaire.

Questionnaire

**Question 1.**

Have you donated blood (blood or plasma) within the last 12 months?

□ Yes	□ No

**Question 1(a).**

If yes to Question 1, what is the frequency of the blood donation?

□ Once in the last 12 months	□ Every few months	□ Monthly	□ Other

Other (details): _______________________________________________

**Question 1(b).**

If yes to Question 1, do you regularly donate blood?

□ Once or More a Year	□ Once Every Several Years	□ Other

Other (details): _______________________________________________

**Question 2.**

Have you or anyone in your family applied stain repellent or water protective coatings to furniture, rugs, carpets, or other textiles such as shoes, boots, or clothing in your home? Example products include: Scotchgard®, Ultra-guard®, TriNova®, Fabric Seal®, or similar products?

□ Yes	□ No	□ Do Not Know

**Question 3.**

What is the primary source of your drinking water in your home?

□ Municipal	□ Bottled	□ Private Well	□ Do Not Know

**Question 4.**

Have you been told by local or federal government officials not to drink your home’s tap water due to PFAS concerns?

□ Yes	□ No

**Question 5.**

Have you installed and/or used a water filtration system certified to remove PFAS from your home’s tap water?

□ Yes	□ No	□ Do Not Know

**Question 6.**

Within the last 12 months, have you used AFFF (firefighting foams) to fight fires or any other related task?

□ Yes	□ No	□ Do Not Know

**Location Section**

**Question 7.**

Please list each city and state that you have lived in over the past 5 years. Include current location.

a. ______________________________________
b. ______________________________________
c. ______________________________________
d. _______________________________________

**Military Experience**

**Question 8.**

Were you involved in firefighting activities while in the military?

□ Yes	□ No

**Question 8(a).**

If yes to Question 8, were the activities within the last 12 months?

□ Yes	□ No

**Question 8(b).**

Did you use AFFF (firefighting foam) to fight fires, for training, or in any other related tasks?

□ Yes	□ No

**Question 8(c).**

Did you use personal protective equipment (PPE) when working with AFFF?

□ Yes	□ No

If yes, mark all that apply.

□ Eye protection	□ Respirator	□ Gloves	□ Coveralls

**Question 8(d).**

Did you have skin contact with AFFF (such as, wading through foam in a hanger)?

□ Yes	□ No

**Question 8(e).**

How many years of firefighting experience did you have?

____________________________________________

**Occupational Experience**

**Question 9.**

Have you worked in civilian firefighting or manufacturing in the past 5 years?

□ Yes	□ No

**Question 9(a).**

If yes to Question 9, did you use personal protective equipment (PPE) when working with stain repellant or water protective coatings?

□ Yes	□ No	□ Not applicable (did not work with stain repellant or water protective coatings)

**_______________________________	______________**

**Signature of Participant**	**Date**
