# Supplementary material for: Use of a capillary blood collection device to monitor exposure to per- and polyfluoroalkyl substances (PFAS) in Veterans living in proximity to potential sources of environmental contamination
Source: J Expo Sci Environ Epidemiol. 2026 Apr 30;36(5):878–87. doi: 10.1038/s41370-026-00884-5 (PMC13538070; doi:10.1038/s41370-026-00884-5)
Supplement: Supplementary file 3 [file 41370_2026_884_MOESM3_ESM.docx]

**Supplemental Methods**

**Materials.** All certified reference materials were purchased from Wellington Laboratories (Guelph, Ontario, Canada), including a mixture of 30 PFAS (PFAC30PAR), a mixture of 24 mass-labeled PFAS (MPFAC-HIF-ES) used as extraction (internal) standards (ES), and a mixture of 7 mass-labeled injection standards (MPFAC-HIF-IS) (IS). Full names, CAS registry numbers and abbreviations for all PFAS measured in this study are shown in **Supplemental Table 1.** High Performance Liquid Chromatography (HPLC)-grade ammonium acetate, isopropanol, and acetonitrile were purchased from Fisher Scientific (Hampton, NH), along with Liquid Chromatography/Mass Spectrometry (LC/MS)-grade methanol and Optima-grade ammonium hydroxide. Fraction V fatty acid-free bovine serum albumin (BSA) was purchased from Sigma-Aldrich (St. Louis, MO). Reverse osmosis (RO) water was produced through a Milli-Q IQ 7000 ultrapure water system. Strata-X-AW 33-μm polymeric weak anion exchange (WAX) 30 mg/well 96-well solid phase extraction plates were purchased from Phenomenex (Torrance, CA). Waters Acquity UPLC BEH C18 (1.7 μm 2.1x50 mm) columns and Waters Acquity UPLC BEH C8 1.7 μm VanGuard pre-columns (2.1x5 mm) were purchased from Waters (Milford, MA). PFAS delay columns (50x2.1 mm) were purchased from Restek (Bellefonte, PA). OneDraw collection devices were acquired from Drawbridge Health (San Diego, CA). Potassium Ethylenediaminetetraacetic acid-EDTA 4 mL Vacutainer tubes were obtained from Fisher Scientific (Becton Dickinson, catalog number BD-367856).

**Quality control.** Each batch of matched plasma and DBS whole blood samples was extracted along with matrix-matched controls, including a matrix blank, low, mid, and high control, prepared for plasma by spiking PFAC30PAR in 4% BSA at concentrations of 0.3, 10, and 20 ng/mL. For DBS controls, 75 μL of each control was spotted onto a OneDraw strip and allowed to dry overnight prior to extraction. Acceptable recoveries were within 50%. A double blank (BKBK) containing only extraction solvents was also included. Both the matrix blank and BKBK were evaluated by comparing their quantifier ion areas under the curve to those of the 0.1 ng/mL calibrator and were deemed acceptable if they were less than 20% of the peak area. External continuous calibration verification (CCV) consisting of 10 ng/mL of all analytes was run every 10 samples to monitor instrument performance. The coefficient of variation (CV) for the CCV samples was deemed acceptable if less than 10%. IS to ES ratios were monitored for overall extraction performance in each batch of 10 samples but were not used to reject any individual samples or quality controls. All calibration curves were within 20% accuracy for the lowest point, 15% for all others, with an r^2^ value >99. Signal/Noise (S/N) for the quantifier ion for all calibrators, quality controls, and samples was required to be greater than or equal to 10 to be reported. All chromatograms were visually inspected for quality and integration. Failure to meet any of these requirements resulted in data being not reported (NR).

**Sample preparation.** All samples and internal standards were brought to room temperature prior to extraction. For each OneDraw sample, the strip carrier was briefly removed from the desiccant cartridge, and one of the two strips was placed in a labeled 2-mL natural polypropylene centrifuge tube using forceps before returning the carrier to the desiccant cartridge (with the unextracted paper strip). For each plasma sample, 50 μL of plasma were aliquoted into a labeled 2-mL natural polypropylene centrifuge tube. Fifty microliters of ES were added to each tube at a concentration of 2 ng/mL for all analytes (excluding the BKBK). The DBS was placed in a labeled 2-mL natural polypropylene centrifuge tube, extracted by adding 300 μL 90:10 acetonitrile in methanol pre-chilled to 4°C, vortexing for 10 min, bath sonicating for 20 min, and centrifuging out precipitated proteins at 4700 RCF for 10 min. The supernatants (approximately 0.3 mL) were transferred to the wells of a 96-deep-well collection plate and dried under nitrogen. Residues were each reconstituted in 600 μL of 5% methanol in RO water and plate vortexed for 10 min.

**Solid phase extraction.** A Phenomenex Strata-X-AW 33-μm polymeric WAX 30 mg/well 96-well solid phase extraction (SPE) plate was placed atop a waste collection reservoir and positioned on a positive pressure manifold. Each well was preconditioned with 1 mL 0.5% ammonium hydroxide in methanol, 1 mL methanol, and 1 mL 10 mM ammonium acetate in RO water. After each of these additions, the liquid was allowed to flow under gravity for 5 min before being pushed onto the sorbent bed, held for 5 min more, and then gently pushed through the bed via positive pressure before each subsequent addition. The samples were then transferred to the SPE plate and washed with 1 mL of 10 mM ammonium acetate in RO water and 1 mL of 40% MeOH following the same timing procedure as described in the preconditioning steps. The sorbent bed was dried for 15 min at 20 PSI. The waste from the reservoir was discarded, and a new 96-deep-well collection plate was placed under the SPE plate. The analytes were then eluted using two 0.9 mL fractions of 0.5% ammonium hydroxide in methanol following the timing procedure above. The collection plate was dried under nitrogen, residue reconstituted in 100 μL of 1 ng/mL IS in 80:20 methanol in RO water, or solvent without IS for BKBK, sealed with a silicone mat, shaken for 15 min, and centrifuged at 1680 RPM for 10 min.

**Calibration curve.** An external calibration curve was prepared by serial dilution using PFAC30PAR in 80:20 methanol in RO water from 0.1-25.6 ng/mL. Mass-labeled internal standard mix MPFAC-HIF-ES was added at 1 ng/mL for most analytes. To determine concentrations of PFAS in the starting sample, we used the actual plasma volume extracted (50 μL) or 75 μL of whole blood for a fully filled DBS, as empirically determined by the OneDraw vendor.

**LC-MS-MS data acquisition.** We adapted our previously reported methods^12-15^ for this analysis**.** UHPLC-coupled electrospray ionization tandem MS was performed with a Shimadzu Nexera LC 40 (Autosampler SIL-40C XS, Pumps LC-40D XS) and data acquired on a Sciex 7500 Q-Trap MS operated in negative ionization mode. An injection of 5 μL of each sample was loaded onto a UPLC BEH C18 column fitted with a UPLC BEHC8 1.7 μm VanGuard 2.1x5 mm pre-column (Waters Acquity). A PFAS delay column was used to separate potential background PFAS in the instrument and mobile phases from the samples^15^. Mobile phases consisted of (A) 10 mM ammonium acetate in RO water and (B) 10 mM ammonium acetate in methanol. The flow rate was 0.4 mL/min, and the gradient was as follows: 0 min, 10% B; 1 min 10% B; 2 min 40% B; 3 min, 40% B; 5 min 50% B; 6 min 60% B; 7 min 60% B; 12 min 80% B; 13 min 90% B; 14 min 10% B. A surrogate blank injection, including valve switching and external needle washing, occurred between samples to reduce carryover. The autosampler was set to perform an external needle rinse with a dip time of 2 s before and after aspiration, to rinse the injection port with 0.5 mL of methanol and to purge the measuring line with 0.1 mL methanol between injections. MS analysis was performed in multiple reaction monitoring mode (MRM) using the instrument settings summarized in **Supplemental Table 2.** Other relevant instrument level parameters were: curtain gas, 40 psi; ion source gas 1, 35 psi; ion source gas 2, 70 psi; ion source temperature, 400 °C; spray voltage,1550V; and CAD gas enabled and set to 6 arbitrary units.

**Data processing and reporting.** We analyzed the instrument level data using Sciex OS with the MQ4 Integration algorithm. Calibration curves were built with linear regression and 1/x weighting. No bottom points were excluded, and a minimum of 5 points were used. All chromatograms were visually inspected and manually integrated, if necessary. To increase accuracy of auto-integration and decrease time manually integrating, we increased peak splitting to between 4-10 points to capture branched and linear isomers together for total calculated concentrations, where applicable. We exported the calculated concentrations of all reportable analytes to Microsoft Excel. For the four analytes with detectable branched and linear isomers, we subtracted the linear isomer from the total to calculate the branched chain concentrations in post-processing.

**Statistical and geospatial analysis.** Linear regression and Spearman’s and Pearson’s correlation analyses were performed in Microsoft Excel to assess within-participant relationships between PFAS concentrations in blood plasma and DBS. Weighted geometric means were calculated for the DBS concentration of each of the measured PFAS analytes and the sum of four target PFAS (PFOS, PFOA, PFHxS, and PFNA) for the full study cohort (N=133). For these analyses, concentrations below the limit of quantification (LOQ) were imputed as a value equal to the LOQ divided by the square root of 2, which is consistent with the methods used by NHANES and other biomonitoring studies^16, 17^ ,and all concentrations were log-transformed prior to analysis.

T-tests and ANOVA were used to assess univariate associations between the transformed analyte concentrations and Veteran demographic characteristics (e.g., sex, race, age at time of blood draw) and PFAS exposure factors (e.g., use of AFFF while in the military, primary source of drinking water, exposure to PFAS-containing coatings, and blood donation in the past 12 months). All demographic characteristics and exposure factors were self-reported. Statistical analyses were performed using SAS Enterprise Guide software, Version 8.3 (SAS Institute Inc., Cary, NC, USA).

County-level geometric mean concentrations of total target PFAS in DBS were visualized on maps using ArcGIS Enterprise software, Version 11.4 (Esri, Redlands, CA, USA). Only Veterans who reported residing in the same county over the previous 5 years were included in the map so that any apparent association between location and measured PFAS could be reasonably attributed to environmental factors specific to the county of residence. Geospatial data on the locations of potential environmental sources of PFAS were retrieved from the Arkansas GIS Office website (https://gis.arkansas.gov). The spatial densities of oil and gas mines and permitted manufacturing facilities tracked by the Arkansas Department of Environmental Quality (DEQ) were calculated and mapped. Point locations of the largest airport (Clinton National Airport, Little Rock, AR) and military installations in the state were also included. Specific layers and transformations included in the map are described in **Supplemental Table 3**.

1.	Sims CR, Sehgal N, Turner D, Havens LA, Morris AJ, Shankar K *et al* Individual and mixtures of PFAS during pregnancy are associated with maternal cardiometabolic outcomes during pregnancy. Environ Health 2025; 24: 26.

2.	Morgan S, Mottaleb MA, Kraemer MP, Moser DK, Worley J, Morris AJ *et al* Effect of lifestyle-based lipid lowering interventions on the relationship between circulating levels of per-and polyfluoroalkyl substances and serum cholesterol. Environ Toxicol Pharmacol 2023; 98: 104062.

3.	Petriello MC, Mottaleb MA, Serio TC, Balyan B, Cave MC, Pavuk M *et al* Serum concentrations of legacy and emerging per- and polyfluoroalkyl substances in the Anniston Community Health Surveys (ACHS I and ACHS II). Environ Int 2022; 158: 106907.

4.	Mottaleb MA, Petriello MC, Morris AJ High-Throughput UHPLC-MS/MS Measurement of Per- and Poly-Fluorinated Alkyl Substances in Human Serum. J Anal Toxicol 2020; 44: 339-347.

16.	Botelho JC, Kato K, Wong LY, Calafat AM Per- and polyfluoroalkyl substances (PFAS) exposure in the U.S. population: NHANES 1999-March 2020. Environ Res 2025; 270: 120916.

17.	Hornung RW, Reed LD Estimation of Average Concentration in the Presence of Nondetectable Values. Applied Occupational and Environmental Hygiene 1990; 5: 46-51.
