# Supplementary material for: Use of a capillary blood collection device to monitor exposure to per- and polyfluoroalkyl substances (PFAS) in Veterans living in proximity to potential sources of environmental contamination
Source: J Expo Sci Environ Epidemiol. 2026 Apr 30;36(5):878–87. doi: 10.1038/s41370-026-00884-5 (PMC13538070; doi:10.1038/s41370-026-00884-5)
Supplement: Supplementary file 4 [file 41370_2026_884_MOESM4_ESM.docx]

**Geospatial Analysis of PFAS exposure.**

**Results.**

Ninety-seven Veterans (72.9% of the study population), who reported residing in the same county in Arkansas over the past five years, were included in a county-level visualization of total target PFAS concentrations in dried whole capillary blood or plasma (**Supplemental Figures 1 and 2**). This subpopulation was distributed across 11 counties, including seven counties with fewer than five participants contributing data to the geographic analysis (Bradley, Chicot, Faulkner, Garland, Lonoke, Pope, and Pulaski). Only one individual was a resident of Chicot County, which is in the southeastern corner of the state. This individual had the highest total target PFAS body burden of those included in this analysis (GM=5.88 ng/mL, 95% CI=1.47-23.6). A resident of Sharp County in northeastern AR also had a high total target PFAS body burden (GM=5.51 ng/mL, 95% CI=1.37-22.1). The Veteran with the lowest total target PFAS body burden resided in St. Francis County in eastern AR (GM=0.61 ng/mL, 95% CI=0.15-2.43). Among the four counties with five or more resident participants, Veterans from Faulkner had the highest average total target PFAS levels (N=11, GM=3.13, 95% CI=2.06-4.76), followed by Pulaski (N=58, GM=2.20 ng/mL, 95% CI=1.83-2.64), Saline (N=15, GM=1.96 ng/mL, 95% CI=1.37-2.80), and Lonoke (N=6, GM=1.47 ng/mL, 95% CI=0.84-2.59). County-level variation in average total target PFAS levels was not statistically significant across the four counties with at least five Veteran participants (p=0.1649).

Of the 11 counties visualized in **Supplemental Figures 1 and 2** six included areas with manufacturing business density greater than two per square mile (Faulkner, Garland, Lonoke, Pope, Pulaski, and Saline). The manufacturing density was greatest in Faulkner and Pulaski Counties. Four counties had areas with at least one oil or gas well per square mile (Bradley, Faulkner, Pope, and White). Pulaski County is also the site of Clinton National Airport (Little Rock) and two military installations (Little Rock Air Force Base and Camp Joseph T. Robinson). Other counties with potential sources of environmental PFAS contamination were not indicated in the Figure because no Veteran participants met the residential criteria for inclusion in the analysis (i.e., having lived only in that county in the past 5 years).

**Discussion**

While larger sample sizes would be needed to identify statistically significant geographic variation, the results in the current study suggest that PFAS body burden in Veterans living in Pulaski County may not be any higher than in Veterans living in adjoining counties, despite the presence of the Little Rock Air Force Base and Clinton National Airport, which are suspected sources of environmental PFAS contamination^1^. The highest PFAS levels observed were in Veterans living in counties in the upper and lower Mississippi Delta regions of Arkansas. While these counties do not have a high density of manufacturing or oil and gas wells, they are highly agricultural areas where pesticide spraying is a potential source of PFAS exposure^2^.

**References:**

1. Sims CR, Sehgal N, Turner D, Havens LA, Morris AJ, Shankar K *et al* Individual and mixtures of PFAS during pregnancy are associated with maternal cardiometabolic outcomes during pregnancy. Environ Health 2025; 24: 26.

2. Mroczko O, Preisendanz HE, Wilson C, Mashtare ML, Elliott HA, Veith TL *et al* Spatiotemporal patterns of PFAS in water and crop tissue at a beneficial wastewater reuse site in central Pennsylvania. J Environ Qual 2022; 51: 1282-1297.

**Figure Legends.**

**Supplemental Figure 1. Geospatial analysis of PFAS exposure in a subset of study subjects using DBS PFAS data.** Geometric means of EPA regulated PFAS levels in OneDraw collected whole capillary blood for the indicated counties are color coded as shown in the legend with known or suspected sites of PFAS contamination including military installations, military and civil aviation facilities and businesses engaged known or suspected industrial uses of PFAS are also shown.

**Supplemental Figure 2. Geospatial analysis of PFAS exposure in a subset of study subjects using plasma PFAS data.** Geometric means of EPA regulated PFAS levels in venous blood plasma for the indicated counties are color coded as shown in the legend with known or suspected sites of PFAS contamination including military installations, military and civil aviation facilities and businesses engaged known or suspected industrial uses of PFAS are also shown.
