# Supplementary material for: Use of a capillary blood collection device to monitor exposure to per- and polyfluoroalkyl substances (PFAS) in Veterans living in proximity to potential sources of environmental contamination
Source: J Expo Sci Environ Epidemiol. 2026 Apr 30;36(5):878–87. doi: 10.1038/s41370-026-00884-5 (PMC13538070; doi:10.1038/s41370-026-00884-5)
Supplement: Supplementary file 5 [file 41370_2026_884_MOESM6_ESM.docx]

| **Target Analyte Name** | **CASRN** | **Abbreviation** |
| --- | --- | --- |
| Perfluorobutanesulfonic acid | 375-73-5 | PFBS |
| Perfluoropentanesulfonic acid | 2706-91-4 | PFPeS |
| Perfluorohexanesulfonic acid | 355-46-4 | PFHxS L |
|  |  | PFHxS Total |
| Perfluoroheptanesulfonic acid | 375-92-8 | PFHpS |
| Perfluorooctanesulfonic acid | 1763-23-1 | PFOS L |
|  |  | PFOS Total |
| Perfluorononanesulfonic acid | 68259-12-1 | PFNS |
| Perfluorodecanesulfonic acid | 335-77-3 | PFDS |
| Perfluorobutanoic acid | 375-22-4 | PFBA |
| Perfluoropentanoic acid | 2706-90-3 | PFPeA |
| Perfluorohexanoic acid | 375-85-9 | PFHxA |
| Perfluoroheptanoic acid | 375-85-9 | PFHpA |
| Perfluorooctanoic acid | 335-67-1 | PFOA |
| Perfluorononanoic acid | 375-95-1 | PFNA |
| Perfluorodecanoic acid | 335-76-2 | PFDA |
| Perfluoroundecanoic acid | 2058-94-8 | PFUdA |
| Perfluorododecanoic acid | 307-55-1 | PFDoA |
| Perfluorotridecanoic acid | 72629-94-8 | PFTrDA |
| Perfluorotetradecanoic acid | 376-06-7 | PFTeDA |
| Hexafluoropropylene oxide dimer acid | 13252-13-6 | HFPO-DA |
| 4,8-Dioxa-3H-perfluorononanoic acid | 919005-14-4 | NaDONA |
| 11-Chloroeicosafluoro-3-oxaundecane-1-sulfonic acid | 763051-92-9 | 11CI-PF3OUdS |
| Perfluorooctanesulfonamide | 754-91-6 | FOSA |
| N-methyl perfluorooctanesulfonamidoacetic acid | 2355-31-9 | N-MeFOSAA L |
|  |  | N-MeFOSAA Total |
| N-ethyl perfluorooctanesulfonamidoacetic acid | 2991-50-6 | N-EtFOSAA L |
|  |  | N-EtFOSAA Total |
| 1H,1H, 2H, 2H-Perfluorohexane sulfonic acid | 757124-72-4 | 4:2 FTS |
| 1H,1H, 2H, 2H-Perfluorooctane sulfonic acid | 27619-97-2 | 6:2 FTS |
| 1H,1H, 2H, 2H-Perfluorodecane sulfonic acid | 39108-34-4 | 8:2 FTS |
| 9-Chlorohexadecafluoro-3-oxanonane-1-sulfonic acid | 756426-58-1 | 9CI-PF3ONS |
| Perfluoro-1-butanesulfonamide | 30334-69-1 | FBSA |
| Perfluoro-1-hexanesulfonamide | 41997-13-**1** | FHxSA |

**Supplemental Table 1. PFAS names, Chemical Abstract Service Registry Numbers, and abbreviations.**
