# Supplementary material for: Use of a capillary blood collection device to monitor exposure to per- and polyfluoroalkyl substances (PFAS) in Veterans living in proximity to potential sources of environmental contamination
Source: J Expo Sci Environ Epidemiol. 2026 Apr 30;36(5):878–87. doi: 10.1038/s41370-026-00884-5 (PMC13538070; doi:10.1038/s41370-026-00884-5)
Supplement: Supplementary file 6 [file 41370_2026_884_MOESM7_ESM.docx]

| ***Group ID*** | ***Compound ID*** | ***Q1 mass*** | ***Q3 mass*** | ***Dwell time (ms)*** | ***EP (V)*** | ***CE*** | ***CXP(V)*** |
| --- | --- | --- | --- | --- | --- | --- | --- |
| PFBS | PFBS | 299 | 80 | 2 | -10 | -65 | -11 |
| PFBS | PFBS_2 | 299 | 99 | 2 | -10 | -35 | -11 |
| PFBS | M3-PFBS_E | 301.9 | 80 | 2 | -10 | -65 | -11 |
| PFPeS | PFPeS | 349.1 | 79.9 | 2 | -10 | -75 | -11 |
| PFPeS | PFPeS_2 | 349.1 | 98.9 | 2 | -10 | -40 | -11 |
| PFHxS | PFHxS L | 398.7 | 79.9 | 2 | -10 | -90 | -11 |
| PFHxS | PFHxS_2 L | 398.7 | 98.8 | 2 | -10 | -75 | -11 |
| PFHxS | PFHxS Total | 398.7 | 79.9 | 2 | -10 | -90 | -11 |
| PFHxS | PFHxS_2 Total | 398.7 | 98.8 | 2 | -10 | -75 | -11 |
| PFHxS | M3_PFHxS_E | 402.1 | 98.8 | 2 | -10 | -90 | -11 |
| Group 1 | M2_PFHxS_I | 403 | 103 | 2 | -10 | -90 | -11 |
| PFHpS | PFHpS | 449 | 79.9 | 2 | -10 | -100 | -11 |
| PFHpS | PFHpS_2 | 449 | 98.8 | 2 | -10 | -85 | -11 |
| PFOS | PFOS L | 498.9 | 79.9 | 2 | -10 | -110 | -11 |
| PFOS | PFOS_2 L | 498.9 | 98.8 | 2 | -10 | -100 | -11 |
| PFOS | PFOS Total | 498.9 | 79.9 | 2 | -10 | -110 | -11 |
| PFOS | PFOS_2 Total | 498.9 | 98.8 | 2 | -10 | -100 | -11 |
| PFOS | M8_PFOS_E | 507.1 | 79.9 | 2 | -10 | -110 | -11 |
| Group 1 | M4_PFOS_I | 502.8 | 79.9 | 2 | -10 | -110 | -11 |
| PFNS | PFNS | 548.8 | 79.9 | 2 | -10 | -120 | -11 |
| PFNS | PFNS_2 | 548.8 | 98.8 | 2 | -10 | -120 | -11 |
| PFDS | PFDS | 599 | 79.9 | 2 | -10 | -130 | -11 |
| PFDS | PFDS_2 | 599 | 98.8 | 2 | -10 | -100 | -11 |
| PFBA | PFBA | 213 | 169 | 2 | -10 | -13 | -13 |
| PFBA | M4_PFBA_E | 217 | 172 | 2 | -10 | -13 | -11 |
| Group 1 | M3_PFBA_I | 216 | 172 | 2 | -10 | -13 | -11 |
| PFPeA | PFPeA | 263 | 219 | 2 | -10 | -12 | -13 |
| PFPeA | M5_PFPeA_E | 268 | 223 | 2 | -10 | -12 | -11 |
| PFHxA | PFHxA | 313 | 269 | 2 | -10 | -12 | -13 |
| PFHxA | PFHxA_2 | 313 | 119 | 2 | -10 | -27 | -11 |
| PFHxA | M5_PFHxA_E | 318 | 273 | 2 | -10 | -12 | -11 |
| Group 1 | M2_PFHxA_I | 315 | 270 | 2 | -10 | -12 | -11 |
| PFHpA | PFHpA | 363 | 319 | 2 | -10 | -14 | -13 |
| PFHpA | PFHpA_2 | 363 | 169 | 2 | -10 | -24 | -11 |
| PFHpA | M4_PFHpA_E | 367 | 322 | 2 | -10 | -14 | -11 |
| PFOA | PFOA | 413 | 369 | 2 | -10 | -15 | -13 |
| PFOA | PFOA_2 | 413 | 169 | 2 | -10 | -24 | -11 |
| PFOA | M8_PFOA_E | 421.1 | 376 | 2 | -10 | -15 | -11 |
| Group 1 | M2_PFOA_I | 417 | 372 | 2 | -10 | -15 | -11 |
| PFNA | PFNA | 463 | 419 | 2 | -10 | -15 | -14 |
| PFNA | PFNA_2 | 463 | 219 | 2 | -10 | -26 | -11 |
| PFNA | M9_PFNA_E | 472.1 | 427 | 2 | -10 | -15 | -11 |
| Group 1 | M5_PFNA_I | 468 | 423 | 2 | -10 | -15 | -11 |
| PFDA | PFDA | 512.9 | 469 | 2 | -10 | -15 | -16 |
| PFDA | PFDA_2 | 512.9 | 219 | 2 | -10 | -29 | -11 |
| PFDA | M6_PFDA_E | 519.1 | 474.1 | 2 | -10 | -15 | -11 |
| Group 1 | M2_PFDA_I | 515.1 | 470.1 | 2 | -10 | -15 | -11 |
| PFUdA | PFUdA | 563 | 519 | 2 | -10 | -16 | -18 |
| PFUdA | PFUdA_2 | 563 | 169 | 2 | -10 | -31 | -11 |
| PFUdA | M7_PFUdA_E | 570 | 525.1 | 2 | -10 | -16 | -11 |
| PFDoA | PFDoA | 613 | 569 | 2 | -10 | -18 | -20 |
| PFDoA | PFDoA_2 | 613 | 319 | 2 | -10 | -36 | -11 |
| PFDoA | M2_PFDoA_E | 615 | 570 | 2 | -10 | -18 | -11 |
| PFTrDA | PFTrDA | 663 | 619 | 2 | -10 | -18 | -20 |
| PFTrDA | PFTrDA_2 | 663 | 168.9 | 2 | -10 | -35 | -11 |
| PFTeDA | PFTeDA | 713 | 669 | 2 | -10 | -20 | -20 |
| PFTeDA | PFTeDA_2 | 713 | 168.9 | 2 | -10 | -40 | -11 |
| PFTeDA | M2_PFTeDA_E | 715 | 670 | 2 | -10 | -20 | -11 |
| HFPO-DA | HFPO-DA | 285 | 169 | 2 | -10 | -11 | -11 |
| HFPO-DA | M3_HFPO-DA_E | 287 | 169 | 2 | -10 | -11 | -11 |
| NaDONA | NaDONA | 377 | 251 | 2 | -10 | -17 | -11 |
| NaDONA | NaDONA_2 | 377 | 85 | 2 | -10 | -35 | -11 |
| 11CI-PF3OUdS | 11CI-PF3OUdS | 630.9 | 450.9 | 2 | -10 | -40 | -11 |
| FOSA | FOSA | 498 | 78 | 2 | -10 | -90 | -11 |
| FOSA | M8_FOSA_E | 506 | 78 | 2 | -10 | -90 | -11 |
| N-MeFOSAA | N-MeFOSAA L | 570 | 419 | 2 | -10 | -27 | -11 |
| N-MeFOSAA | N-MeFOSAA Total | 570 | 419 | 2 | -10 | -27 | -11 |
| N-MeFOSAA | d3-MeFOSAA_E | 573 | 419 | 2 | -10 | -27 | -11 |
| N-EtFOSAA | N-EtFOSAA L | 584 | 419 | 2 | -10 | -30 | -11 |
| N-EtFOSAA | N-EtFOSAA Total | 584 | 419 | 2 | -10 | -30 | -11 |
| N-EtFOSAA | d5-EtFOSAA_E | 589 | 419 | 2 | -10 | -30 | -11 |
| 4:2 FTS | 4:2 FTS | 327 | 307 | 2 | -10 | -27 | -11 |
| 4:2 FTS | M2-4:2FTS_E | 329 | 309 | 2 | -10 | -27 | -11 |
| 6:2 FTS | 6:2 FTS | 427 | 407 | 2 | -10 | -32 | -11 |
| 6:2 FTS | M2-6:2FTS_E | 429 | 409 | 2 | -10 | -32 | -11 |
| 8:2 FTS | 8:2 FTS | 527 | 507 | 2 | -10 | -37 | -11 |
| 8:2 FTS | M2-8:2FTS_E | 529 | 509 | 2 | -10 | -42 | -11 |
| 9CI-PF3ONS | 9CI-PF3ONS | 531 | 351 | 2 | -10 | -37 | -11 |
| FBSA | FBSA | 298 | 78 | 2 | -10 | -27 | -11 |
| FHxSA | FHxSA | 398 | 78 | 2 | -10 | -27 | -11 |

**Supplemental Table 2. MRM masses and instrument settings for native PFAS, Internal and External Standards.**
