# Supplementary material for: Use of a capillary blood collection device to monitor exposure to per- and polyfluoroalkyl substances (PFAS) in Veterans living in proximity to potential sources of environmental contamination
Source: J Expo Sci Environ Epidemiol. 2026 Apr 30;36(5):878–87. doi: 10.1038/s41370-026-00884-5 (PMC13538070; doi:10.1038/s41370-026-00884-5)
Supplement: Supplementary file 7 [file 41370_2026_884_MOESM8_ESM.docx]

| **Layer** | **Transformation** | **Data Source** |
| --- | --- | --- |
| Military Installations | None | Arkansas Army National Guard. *Military Installation Area.* Washington, D.C.: U.S. Department of Defense [producer]. Little Rock, A.R.: State of Arkansas [distributor]. 2017. Retrieved from: [https://gis.arkansas.gov/product/military-installation-area-polygon/](https://urldefense.com/v3/__https:/gis.arkansas.gov/product/military-installation-area-polygon/__;!!LFqOYw!uCOpicpOGjSo2QLWRpe0nYbyLqXNhO4hYrdXkcSaXqwSwKZGwAJ96RIhwtktlQTsKcpA1KD4rI_1ZkfihK3i3Kopomk$) Access date: April 29, 2025 |
| Environmental Facilities | Calculated density of manufacturing businesses (point) per square mile, displayed density (polygon) | Arkansas Department of Environmental Quality. *Permit Data System.* Little Rock, A.R.: State of Arkansas, 2017. Retrieved from: [https://gis.arkansas.gov/product/enviromental-facility-point/](https://urldefense.com/v3/__https:/gis.arkansas.gov/product/enviromental-facility-point/__;!!LFqOYw!uCOpicpOGjSo2QLWRpe0nYbyLqXNhO4hYrdXkcSaXqwSwKZGwAJ96RIhwtktlQTsKcpA1KD4rI_1ZkfihK3ijYi20X4$) Access date: May 1, 2025 |
| County Boundaries | Joined with county-level geometric mean target PFAS values | Arkansas GIS Office. County Boundaries of Arkansas. Little Rock, A.R.: State of Arkansas, 2020. Retrieved from: [https://gis.arkansas.gov/product/county-boundary-polygons/](https://urldefense.com/v3/__https:/gis.arkansas.gov/product/county-boundary-polygons/__;!!LFqOYw!uCOpicpOGjSo2QLWRpe0nYbyLqXNhO4hYrdXkcSaXqwSwKZGwAJ96RIhwtktlQTsKcpA1KD4rI_1ZkfihK3iHapMkgg$) Access date: April 29, 2025 |
| Permitted Oil and Gas Wells | Calculated density of wells (point) per square mile, displayed density (polygon) | Arkansas Oil and Gas Commission. *Arkansas Oil and Gas Wells.* Little Rock, A.R.: State of Arkansas, 2024. Retrieved from: [https://gis.arkansas.gov/product/arkansas-oil-and-gas-wells/](https://urldefense.com/v3/__https:/gis.arkansas.gov/product/arkansas-oil-and-gas-wells/__;!!LFqOYw!uCOpicpOGjSo2QLWRpe0nYbyLqXNhO4hYrdXkcSaXqwSwKZGwAJ96RIhwtktlQTsKcpA1KD4rI_1ZkfihK3iDLo4_I4$) Access date: April 30, 2025 |
| Airports | Filtered to display Little Rock Airport (point) only | Federal Aviation Administration. Airport Data and Information Portal. Washington, D.C.: U.S. Department of Transportation [producer]. Little Rock, A.R.: State of Arkansas [distributor]. 2024. Retrieved from: [https://gis.arkansas.gov/product/airport-point/](https://urldefense.com/v3/__https:/gis.arkansas.gov/product/airport-point/__;!!LFqOYw!uCOpicpOGjSo2QLWRpe0nYbyLqXNhO4hYrdXkcSaXqwSwKZGwAJ96RIhwtktlQTsKcpA1KD4rI_1ZkfihK3iGFKcYfk$) Access date: April 30, 2025 |
| Basemap | None | Esri. "Human Geography Base" [basemap]. Scale Not Given. "Human Geography Map (World Edition) ". Retrieved from: [https://www.arcgis.com/home/item.html?id=2afe5b807fa74006be6363fd243ffb30](https://urldefense.com/v3/__https:/www.arcgis.com/home/item.html?id=2afe5b807fa74006be6363fd243ffb30__;!!LFqOYw!uCOpicpOGjSo2QLWRpe0nYbyLqXNhO4hYrdXkcSaXqwSwKZGwAJ96RIhwtktlQTsKcpA1KD4rI_1ZkfihK3iGIZ6vao$). Access date: May 22, 2025. |

**Supplemental Table 3 Data sources for geospatial analysis.**
